# Idiopathic Localized Acquired Cutis Laxa in an Adult Male: A Case Report

**DOI:** 10.7759/cureus.100626

**Published:** 2026-01-02

**Authors:** Ahmed F Alanazi, Ahmed Alharbe, Rayan H Asiree, Haifa M Al-Shammari, Mohammad F Munshi

**Affiliations:** 1 College of Medicine, Imam Mohammed Ibn Saud Islamic University, Riyadh, SAU; 2 College of Medicine, King Saud Bin Abdulaziz University for Health Sciences, Riyadh, SAU; 3 College of Medicine, Majmaah University, Majmaah, SAU; 4 Pathology, Regional Laboratory, King Saud Medical Complex, Riyadh, SAU; 5 Dermatology, King Salman Hospital, Riyadh First Health Cluster, Riyadh, SAU

**Keywords:** acquired cutis laxa, adult-onset cutis laxa, congenital cutis laxa, cutis laxa, skin laxity

## Abstract

Cutis laxa (CL) is a rare connective-tissue disorder characterized by loose, inelastic skin due to defects in elastic fiber production or structure. Acquired cutis laxa (ACL) typically develops in adulthood and may follow inflammatory or immune-mediated events, though idiopathic cases remain uncommon.

We report a 29-year-old male with a 10-year history of progressive skin laxity affecting the face, neck, and upper back. There were no preceding infections, drug exposures, or inflammatory skin conditions. Medical, surgical, and family histories were unremarkable. Examination revealed redundant, wrinkled skin with markedly reduced recoil in the involved areas, without joint hypermobility or systemic features suggestive of connective-tissue disease. Laboratory tests and imaging were normal. Histopathology demonstrated diminished and fragmented elastic fibers throughout the dermis, confirmed by Verhoeff-Van Gieson staining, consistent with ACL.

This case represents an idiopathic, localized adult-onset form of ACL. The characteristic histological findings, along with the absence of systemic involvement, support the diagnosis. It is important to distinguish this condition from others, such as Ehlers-Danlos syndrome, anetoderma, and pseudoxanthoma elasticum, since overlapping clinical features can lead to misdiagnosis. Idiopathic ACL is rare and often under-recognized, and this case represents the first reported regional idiopathic instance, to the best of our knowledge, from Saudi Arabia. Overall, this case highlights the value of careful clinical assessment and histological evaluation in patients with slowly progressive, non-inflammatory skin laxity.

## Introduction

Cutis laxa is an uncommon connective-tissue disorder characterized by loose, sagging skin with poor elasticity, resulting from abnormalities in the quantity or structure of elastic fibers within the dermis. The condition may present at birth or arise later in life, and it can involve only the skin or extend to multiple organ systems [1-5].

Inherited forms of cutis laxa demonstrate significant genetic variability, with autosomal dominant, autosomal recessive, and X-linked patterns all described in the literature. These congenital variants often show systemic involvement affecting structures such as the lungs, cardiovascular system, gastrointestinal tract, and genitourinary tract. Acquired cutis laxa is far less common and typically develops following inflammatory, infectious, or immune-mediated skin processes. Research has shown that despite the clinical appearance of elastin loss, some acquired forms may paradoxically display increased elastin production at the molecular level. However, these fibers fail to assemble properly into functional elastic fibers. This suggests that both degradation and impaired fiber formation play important roles in the pathogenesis of the acquired subtype [4]. Given the wide clinical variability and the incomplete understanding of its underlying mechanisms, each newly documented case of cutis laxa adds to the collective knowledge of its presentation and pathology. Treatment is largely supportive; surgical reconstruction (excision, rhytidectomy, blepharoplasty) has shown the most consistent cosmetic benefit in reported cases, while medical therapies and topical measures generally offer limited improvement [2,6].

In this report, we present a case of Cutis laxa in a 29-year-old male, specifically acquired cutis laxa, given the late onset and lack of family history. This case is presented due to its rarity, the diagnostic challenge it posed, and its regional relevance. We will discuss the clinical and histopathological features that contribute to better recognition of this rare condition.

## Case presentation

**Figure 1:**
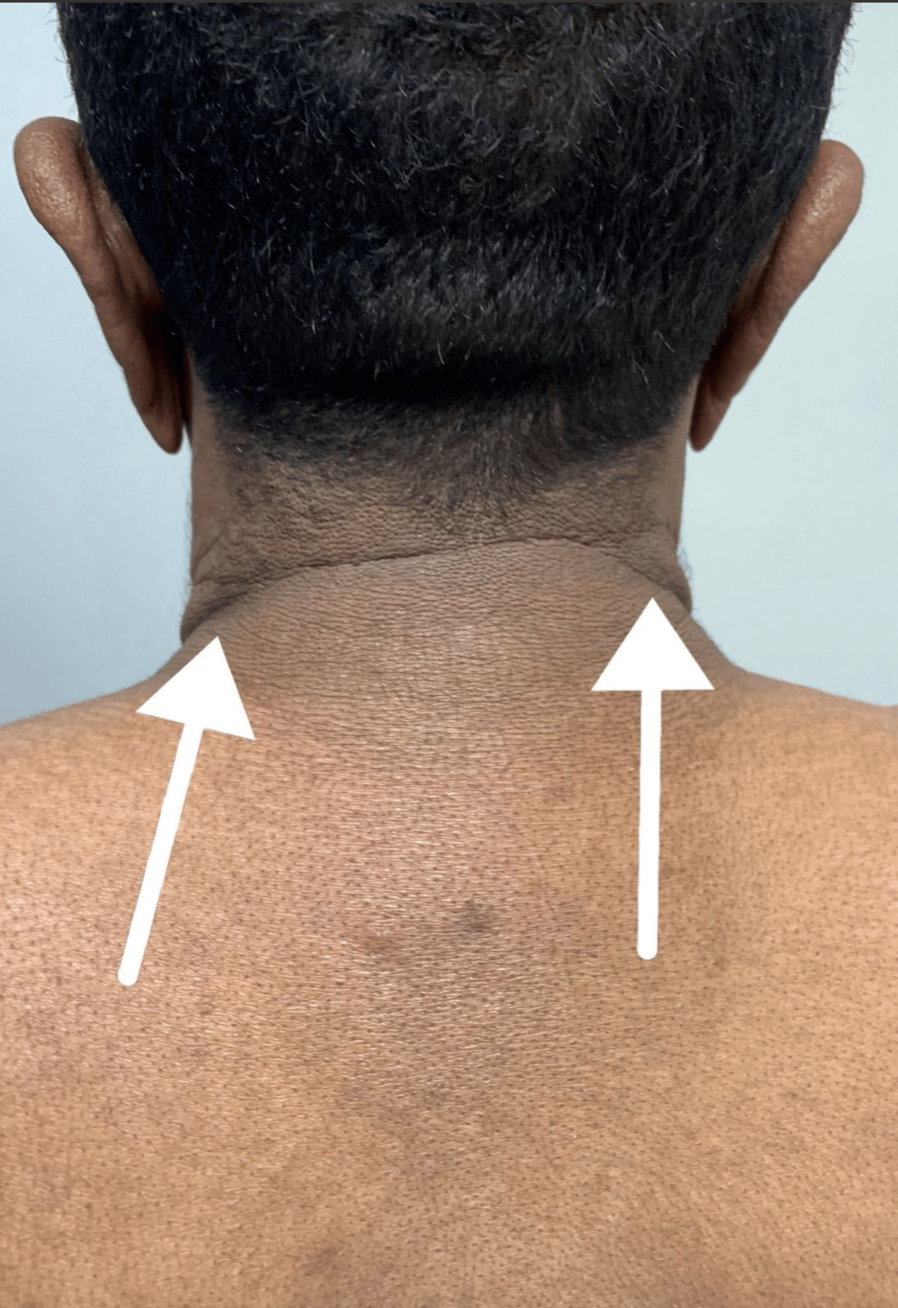
Clinical image. Lax redundant skin of the neck.

**Figure 2:**
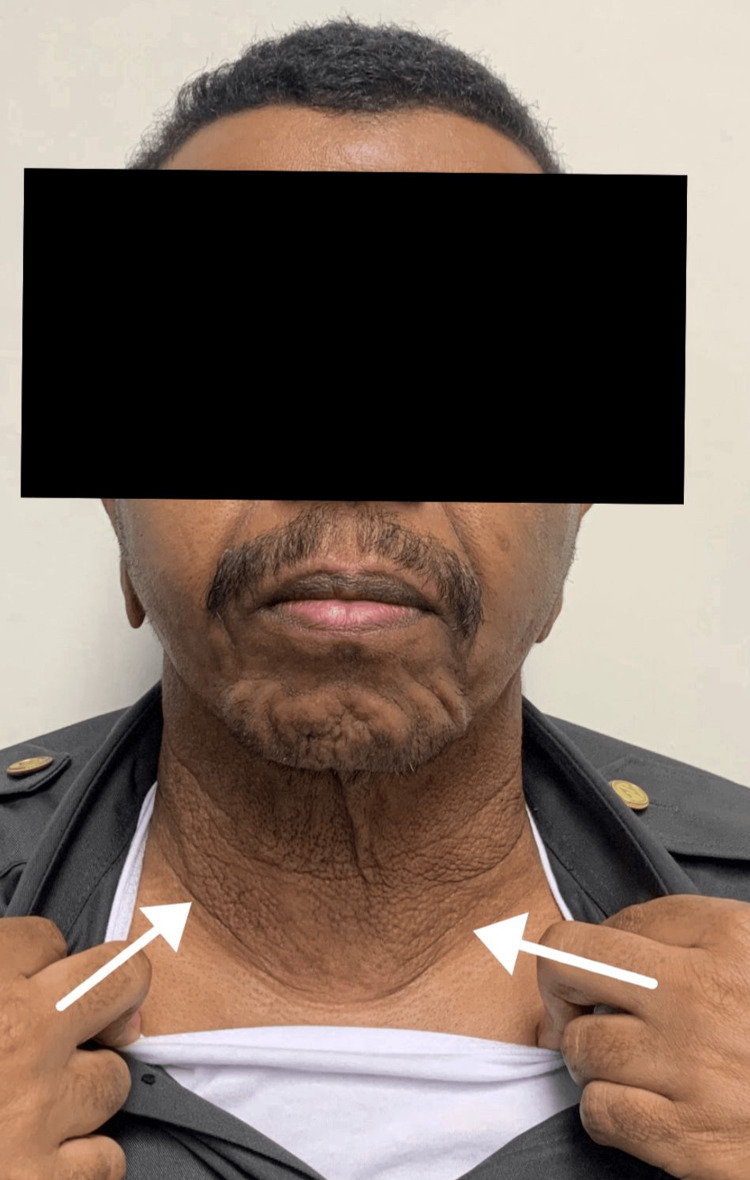
Clinical image. Redundant and markedly wrinkled skin is visible on the neck and face of the patient.

We present the case of a 29-year-old male with no past medical history who presented to our clinic with a 10-year history of progressive loosening and wrinkling of the skin of the face, neck, and upper back, while other areas remained unaffected (Figures 1, 2).

The patient first noticed the lesion in his late teens to early twenties, and it slowly progressed over time. His sole concern was cosmetic appearance, and he reported no pain, pruritus, ulceration, or other local symptoms. He sought medical care at multiple clinics over the years without receiving a definitive diagnosis or effective treatment. The patient’s past medical history was unremarkable. He had no known chronic or autoimmune disease, no prior surgeries, no allergies, and took no regular medications. He denied tobacco use, recreational drugs, and any systemic symptoms such as weight loss, fever, headache, dyspnea, or gastrointestinal complaints. There was no family history of similar conditions.

On examination, the patient appeared older than his chronological age. The skin of the face, neck, and upper back was lax, redundant, and markedly wrinkled, with reduced recoil but without active inflammation, scale, or induration. The changes were most pronounced centrally on the anterior neck. Ophthalmologic, cardiovascular, respiratory, and neurological examinations were otherwise normal, with no stigmata of systemic connective tissue disorder.

Laboratory investigations, including complete blood count, liver function tests, kidney function tests, and antinuclear antibodies, were unremarkable. Chest radiograph and urine analysis showed no abnormalities. Ophthalmic evaluation was also unremarkable.

**Figure 3:**
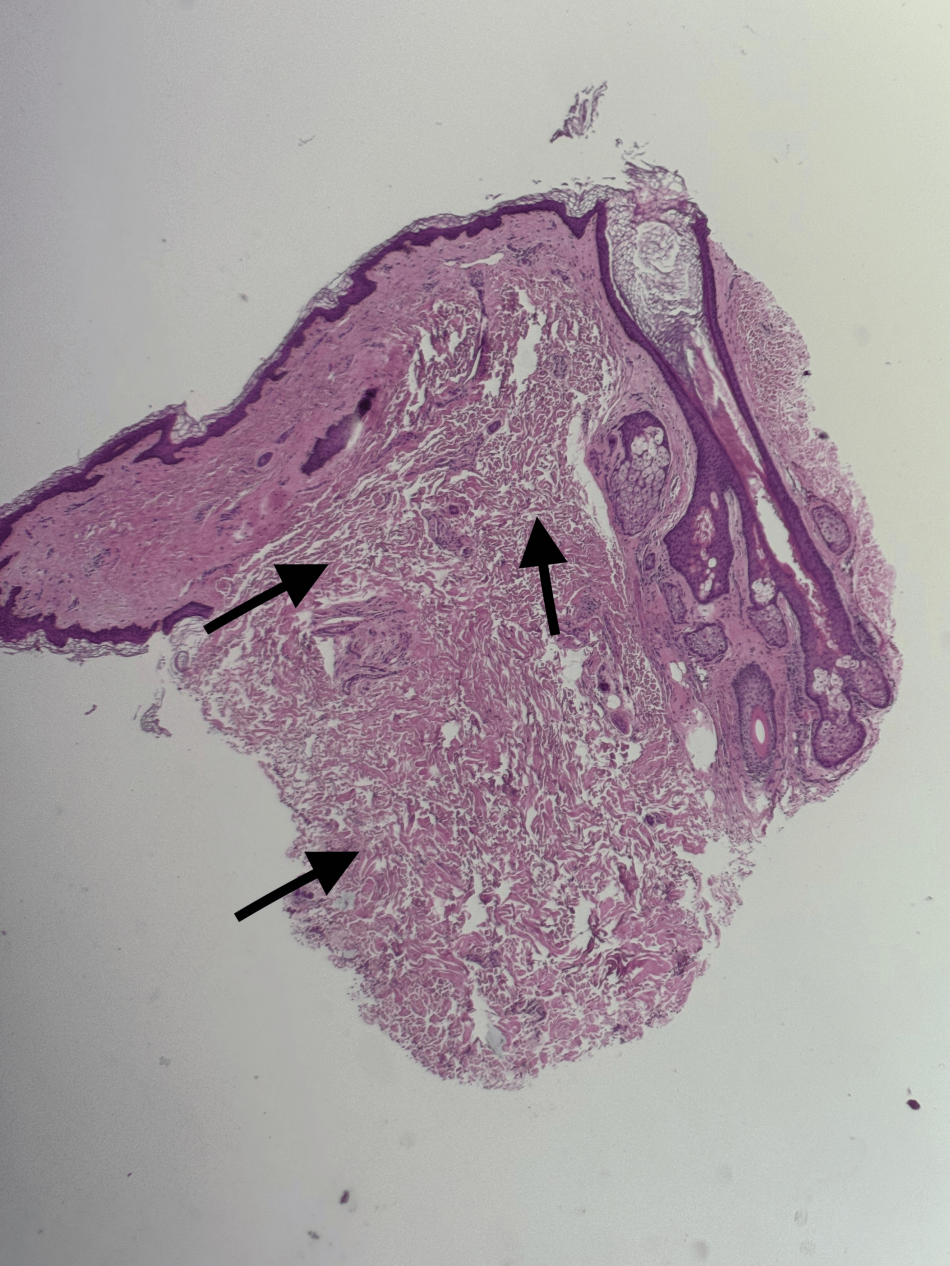
Histology findings from the biopsy site The section shows an unremarkable epidermis. The dermis demonstrates marked reduction and fragmentation of elastic fibers, resulting in loose, lax, and redundant connective tissue (arrows). VVG staining highlights the near-complete absence of elastic fibers, supporting the diagnosis of cutis laxa. H&E: hematoxylin and eosin; VVG: Verhoeff–Van Gieson. Original magnification ×10.

A punch biopsy was taken from an affected area of the neck. Histopathology demonstrated an unremarkable epidermis. The dermis showed marked reduction and fragmentation of elastic fibers, producing a loose and redundant connective tissue appearance (Figure 3).

**Figure 4:**
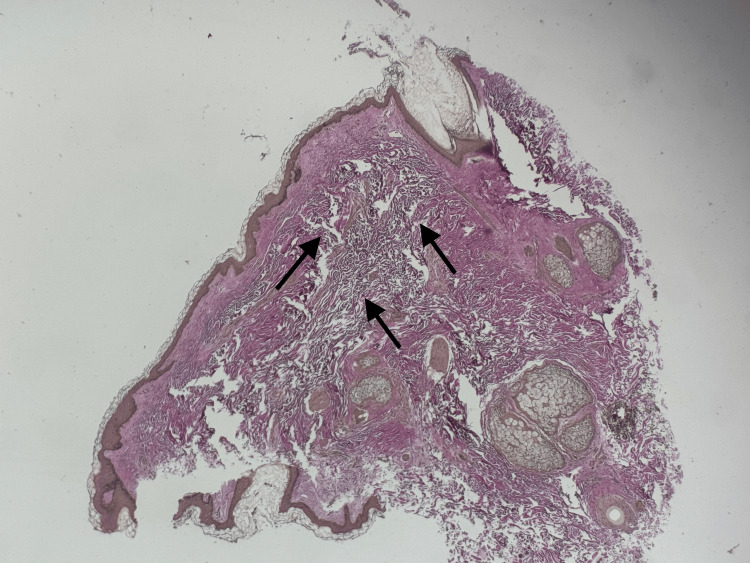
Histology findings from biopsy site. On Verhoeff-Van Gieson (VG) special staining, there is marked to near-complete absence of elastic fibers within the dermis (arrows), supporting the diagnosis of cutis laxa. Original magnification ×10.

Verhoeff-Van Gieson (VVG) special stain highlighted a significant to near-complete absence of elastic fibers in the sampled dermis, consistent with the diagnosis of cutis laxa (Figure 4).

## Discussion

Acquired cutis laxa (ACL) is an extremely rare connective-tissue disorder, with fewer than 100 adult-onset cases reported worldwide. It is characterized by progressive skin laxity due to elastic fiber abnormalities [1-4]. Most cases follow inflammatory, infectious, or drug-induced triggers, though idiopathic forms are uncommon [3,4]. Localized presentations are rarer than generalized forms and typically lack systemic involvement [3-9]. To date, no idiopathic, localized ACL cases have been reported from Saudi Arabia.

Our patient presented with slowly progressive laxity of the face, neck, and upper back beginning in early adulthood, a pattern consistent with adult-onset ACL. Similar to previously reported cases, the absence of pain, pruritus, or systemic symptoms helped distinguish this disorder from other connective-tissue diseases [1,6]. Localized or regional presentations have been described in the literature, such as abdominal involvement [7], acral forms [8], or facial-predominant types associated with hematologic disease [9]. In comparison, our case represents a regional distribution affecting cosmetically significant areas, without evidence of systemic illness, laboratory abnormalities, or underlying malignancy.

Histopathology remains essential for diagnosis. Our findings of marked reduction and fragmentation of elastic fibers, highlighted by Verhoeff-Van Gieson staining, are consistent with previously reported cases. While some acquired cases show increased elastin gene expression at the molecular level, the failure of these fibers to assemble properly contributes to the paradox of increased elastin production but profound clinical laxity [4].

The pathogenesis of ACL is incompletely understood. Proposed mechanisms include excessive degradation of elastic fibers by elastases, aberrant elastin turnover, immune-mediated injury, and post-inflammatory elastolysis [4,6]. Certain cases have been linked to preceding inflammatory eruptions, infections, drug reactions, or autoimmune conditions [1,3,8]. Our patient denied any such history, reflecting the idiopathic subset of ACL in which no trigger can be identified. Retrospective reviews show that a significant proportion of adult patients in this subset had no discernible inciting event [3].

A crucial component of the diagnostic work-up is distinguishing ACL from clinically similar disorders. In our patient, PXE was excluded because it typically presents with yellow papules and calcified elastic fibers, which were absent both clinically and histologically. Ehlers-Danlos syndrome was also ruled out, as it features hyperextensible skin and joint hypermobility, whereas ACL shows loose skin with decreased recoil and no vascular fragility. Other conditions, including weight-loss-related skin redundancy, mid-dermal elastolysis, and congenital cutis laxa, were excluded based on clinical examination and history.

Management of ACL remains challenging. Medical therapies, including vitamin supplementation and topical emollients, have shown minimal or no sustained benefit in reported cases [1,6]. In contrast, multiple reports describe satisfactory cosmetic outcomes after surgical excision, rhytidectomy, blepharoplasty or flap reconstruction, although recurrence and the need for revision surgery have been noted in some patients [2]. Conservative treatment with vitamin E and emollients has likewise shown little improvement in selected cases [6]. Therefore, surgical correction remains the most effective option for patients with stable, localized or cosmetically significant disease, as in our case [2,7]. Less invasive approaches such as botulinum toxin have been proposed, but supporting evidence remains limited and long-term outcomes are unclear [6,10].

## Conclusions

This case underscores the importance of considering ACL in adults with progressive, non-inflammatory skin laxity, especially when systemic causes remain unexplained. Biopsy with elastic fiber staining is crucial for confirming the diagnosis and distinguishing ACL from similar disorders. Early recognition can prevent diagnostic delays, as seen in our patient, who sought care at multiple clinics without a definitive diagnosis. Given the rarity of ACL and its diverse presentation, this case contributes to the growing understanding of its etiology and clinical spectrum. Increased clinical awareness and further research into therapeutic approaches are essential for improving patient outcomes.
